# Mass modeling of fig (*Ficus carica* L.) fruit with some physical characteristics

**DOI:** 10.1002/fsn3.20

**Published:** 2012-12-25

**Authors:** Feizollah Shahbazi, Satar Rahmati

**Affiliations:** Faculty of Agriculture, Lorestan UniversityKhorramabad, Iran

**Keywords:** Fig, physical characteristics, mass prediction

## Abstract

Horticultural crops with the similar weight and uniform shape are in high demand in terms of marketing value, which are used as food. For proper design of grading systems, important relationships among the mass and other properties of fruits such as length, width, thickness, volumes, and projected areas must be known. The aim of this research was to measure and present some physical properties of fig fruits. In addition, Linear, Quadratic, S-curve, and Power models are used for mass predication of fig fruits based on measured physical properties. The results showed that all measured physical properties were statistically significant at the 1% probability level. For mass predication of fig fruits, the best and the worst models were obtained based on criteria projected area and thickness of the fruits with determination coefficients (*R*^2^) of 0.984 and 0.664, respectively. At last, from economical standpoint, mass modeling of fig fruits based on first projected area is recommended.

## Introduction

The fig (*Ficus carica* L.) fruit is one of the favorite dried fruits in the world. This horticultural product is grown mostly in Iran, Turkey, and Afghanistan, and it has been one of the important nonoil agricultural export commodities in the last three decades in Iran. The total annual fig production of Iran was about 88,000 tons in 2007 (FAOSTAT 2009). It is widely used in confectionery, snack foods, and pastry industries (Doymaz 2005).

Physical characteristics of agricultural materials and their relationships are necessary for the design of some postharvest processing systems, such as handling, sorting, and packaging. Among these properties, mass, dimensions, volume, and projected areas are the most important factors (Mohsenin 1986). Consumers prefer fruits with equal weight and uniform shape. Mass grading of fruit can reduce packaging and transportation costs by providing accurate method of automatic classification and optimum packaging configuration (Peleg^1985^). Classification of fruits is often done based on their mass, size, volume, and projected areas. Using the electrical grading system is more complex and expensive and mechanical systems work slowly. Therefore, developing a grading system that grades fruits based on their mass may be more economical. Mass classification of more fruits is the most accurate automatic classification. Therefore, determining the relationships among mass and dimensions, volumes, and projected areas can be useful and applicable (Khoshnam et al. 2007).

A number of studies have been conducted on the mass modeling of fruits based on their physical properties. Tabatabaeefar et al. (2000) developed 11 models based on dimensions, volumes, and surface areas for mass predication of orange fruits. Al-Maiman and Ahmad (2002) studied the physical properties of pomegranate and found models for predicting fruit mass while employing dimensions, volume, and surface areas. A Quadratic model (*M =* 0.08*c*^2^ *+* 4.74*c* *+* 5.14, *R*^2^ *=* 0.89), to calculate the apple mass based on its minor diameter, was determined by Tabatabaeefar and Rajabipour (2005). Mass models for Iranian kiwi fruit based on the fruit dimensions, volumes, and projected areas were determined by Lorestani and Tabatabaeefar (2006). In addition, they found that the intermediate diameter was more appropriate to estimate the mass of kiwi fruit. Khanali et al. (2007) determined similar mass models for tangerine fruit. Also, Naderi-Boldaji et al. (2008) used this method for predicting the mass of apricot fruit. They found a nonlinear equation (*M* = 0.0019*c*^2.693^, *R*^2^ = 0.96) between apricot mass and its minor diameter. Some researchers (Fadavi et al. 2005; Kingsly et al. 2006) reported mass models for pomegranate fruit. Lorestani and Ghari (2012) concluded that the best model for mass prediction of Fava bean among dimensional models was Linear based on width and Power form based on third projected area perpendicular to *L* direction of bean.

No detailed studies concerning mass modeling of fig fruit have yet been performed. The aims of this study were to determine the most suitable model for predicting fig fruit mass by its physical attributes and specify some physical properties of Iranian fig fruit to form an important database for other researches.

## Materials and Methods

**Figure 1:**
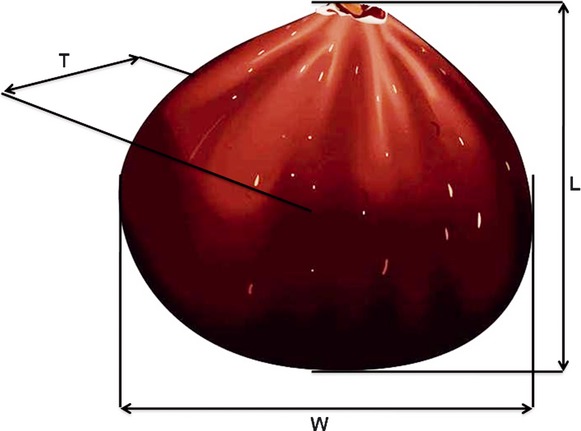
Dimensional characteristics of black fig fruit: *L*, length; *W*, width; *T*, thickness.

Fresh-harvested fig fruits from Siah Lorestan cultivar, which were obtained from Lorestan province, Iran, on August 2012, were used in this study. In order to determine the physical properties, 150 fig fruits were randomly selected. Selected samples were healthy and free from any injuries. Samples of fruits were weighed and dried in an oven at a temperature of 78°C for 48 hours, and then, weight loss on drying to a final constant weight was recorded as moisture content. The mass of each fig fruit (*M*) was measured using a digital balance with an accuracy of 0.01 g. For each fig fruit, three linear dimensions were measured by using a digital caliper with accuracy of 0.01 mm, including length (*L*), width (*W*), and thickness (*T*; Fig. 1). Water displacement method was used for determining the measured volume (*V*_m_) of fruits. Fruits geometric mean diameter (*D*_g_) and surface area (*S*) were determined as suggested by Mohsenin (1986):


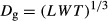
(1)


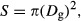
(2)

where *S* is fruit surface area (mm^2^), and *D*_g_ is geometric mean diameter (mm). In addition, fruit average projected areas perpendicular to dimensions (*PA*_1_, *PA*_2_, and *PA*_3_) were measured by a ΔT area-meter, MK2 model, device with an accuracy of 10 mm^2^, and then, the criteria projected area (*CPA*) was calculated as suggested by Mohsenin (1986):


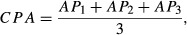
(3)

where *PA*_1_ (perpendicular to *L* direction of fruit), *PA*_2_ (perpendicular to *T* direction of fruit), and *PA*_3_ (perpendicular to *W* direction of fruit) are first, second, and third projected areas (mm^2^), respectively.

The following models are considered for estimating the mass models for fig fruits:

- Single variable regression of fig fruit mass based on fruit dimensional properties including length (*L*), width (*W*), thickness (*T*), and geometric mean diameter (*D*_g_).
- Single or multiple variable regression of fig fruit mass based on fruit projected areas (*PA*_1_, *PA*_2_, and *PA*_3_), surface area (*S*), and *CPA*.
- Single regression of fig fruit mass based on measured volume (*V*_m_), volume of the fruit assumed as oblate spheroid shape (*V*_osp_), and volume of the fruit assumed as ellipsoid shape (*V*_ellip_).

According to the third model to achieve the fig fruit mass based on volumes, three volume values were either measured or calculated. First, measured volume (*V*_m_) was measured, and then the fig fruit shape was assumed as a regular geometric shape, that is, oblate spheroid (*V*_osp_) and ellipsoid (*V*_ellip_) shapes, and their volume was thus calculated as follows:


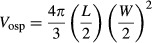
(4)


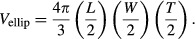
(5)

Four models including Linear, Quadratic, S-curve, and Power models were used for mass predication of fig fruits based on measured physical properties, as represented in the following expressions, respectively:


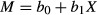
(6)


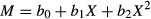
(7)


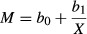
(8)


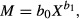
(9)

where *M* is mass (g), *X* is the value of a parameter (physical characteristics) that we want to find its relationship with fruit mass, and *b*_0_, *b*_1_, and *b*_2_ are curve-fitting constants. The coefficient of determination (*R*^2^) value was used for evaluating the goodness of fit. In general, for regression equations, the *R*^2^ value near to 1.00 shows the better fit (Stroshine 1998). Analyzing the data and finding the fig fruit mass models based on measured physical properties were done using SPSS 15 software (SPSS Inc, Chicago, IL).

## Results and Discussion

### Physical properties of fig fruit

Table 1 shows the measured physical properties of studied fig fruits. The properties are measures at the moisture content of about 81.13% wet basis. As seen from the data in Table 1, the effects of the all properties, on the mass of fig fruit, are statistically significant at 1% probability level. The mean values of measured physical properties of studied fig fruits (length, width, thickness, geometric mean diameter, surface area, mass, first projected area, second projected area, third projected area, *CPA*, measured volume, oblate spheroid volume, and ellipsoid shapes volume) were 32.072 mm, 43.086 mm, 40.179 mm, 38.088 mm, 4601.427 mm^2^, 35.433 g, 1383.533 mm^2^, 1370.066 mm^2^, 1127.866 mm^2^, 1293.922 mm^2^, 46933.332 mm^3^, 32230.019 mm^3^, and 29821.124 mm^3^, respectively.

**Table 1:** Some physical properties of fig fruit (at 81.13% w.b. moisture content)

	Value			Significant level (*P*)
	
Properties	Average	Maximum	Minimum	
*L* (mm)	32.072	37.86	27.78	<0.01
*W* (mm)	43.086	54.98	35.22	<0.01
*T* (mm)	40.179	52.80	33.39	<0.01
*D*_g_ (mm)	38.088	47.90	32.42	<0.01
*S* (mm^2^)	4601.427	7204.58	3302.16	<0.01
*M* (g)	35.433	69.10	20.21	<0.01
*AP*_1_ (mm^2^)	1383.533	2217.13	898.22	<0.01
*AP*_2_ (mm^2^)	1370.066	2317.21	828.13	<0.01
*AP*_3_ (mm^2^)	1127.966	1786.24	767.14	<0.01
*CPA* (mm^2^)	1293.822	2106.66	849.12	<0.01
*V*_m_ (mm^3^)	46933.332	100002.28	25019.18	<0.01
*V*_osp_ (mm^3^)	32230.019	59891.95	18825.87	<0.01
*V*_ellip_ (mm^3^)	29821.124	57517.19	17847.69	<0.01

### Mass modeling

Table 2 shows the obtained best models and their coefficient of determination (*R*^2^) for mass predication of fig fruits based on the measured physical properties. The results of the *F*-test and *T*-test in SPSS 15 software showed that all the coefficients of the models were significant at the 1% probability level.

**Table 2:** The best models for mass prediction of fig fruit with some physical characteristics

Dependent variable (g)	Independent variable	The best-fitted model	Constant parameters			
	
			*b*_0_	*b*_1_	*b*_2_	*R*^2^
*M*	*L* (mm)	Quadratic	506.085	−32.832	0.562	0.785
*M*	*W* (mm)	Quadratic	58.443	−3.318	0.064	0.969
*M*	*T* (mm)	Quadratic	194.232	−9.468	0.135	0.664
*M*	*D*_g_ (mm)	Quadratic	−34.933	0.524	0.034	0.908
*M*	*AP*_1_ (mm^2^)	Quadratic	5.881	0.009	8.619 × 10^−6^	0.973
*M*	*AP*_2_ (mm^2^)	Quadratic	5.327	0.013	5.995 × 10^−6^	0.957
*M*	*AP*_3_ (mm^2^)	Quadratic	9.848	0.001	1.859 × 10^−5^	0.970
*M*	*CPA* (mm^2^)	Quadratic	2.930	0.014	8.221 × 10^−6^	0.984
*M*	*S* (mm^2^)	Linear	−24.591	0.013	–	0.908
		Quadratic	−27.991	0.001	−1.344 × 10^−7^	0.908
*M*	*V*_m_ (mm^3^)	Quadratic	−6.557	0.001	−2.998 × 10^−9^	0.938
*M*	*V*_osp_ (mm^3^)	Linear	−1.969	0.001	–	0.965
*M*	*V*_ellip_ (mm^3^)	Quadratic	−12.075	0.002	−7.576 × 10^−9^	0.908

#### Modeling based on dimensions

The results of mass modeling of fig fruit based on the dimensional characteristics, including length (*L*), width (*W*), thickness (*T*), and geometric mean diameter (*D*_g_), showed that Quadratic model based on width (*W*) had the highest *R*^2^ value among the others (Table 2) and we have:


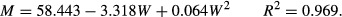
(10)

In addition, Quadratic model can predict the relationships between the mass with length (*L*) and thickness (*T*) with *R*^2^ values of 0.785 and 0.664, respectively. Therefore, mass modeling of fig fruit based on width is recommended. Similar model (nonlinear) was suggested by Tabatabaeefar et al. (2000) for mass predication of orange fruit mass based on fruit width. Their recommended model was *M* = 0.069*b*^2^ − 2.95*b* − 39.15, *R*^2^ = 0.97. In addition, 11 models for predicting mass of apples based on geometrical attributes were recommended by Tabatabaeefar and Rajabipour (2005). They recommended an equation for calculating apple mass based on minor diameter as *M* = 0.08*c*^2^ − 4.74*c* + 5.14, *R*^2^ = 0.89. Ghabel et al. (2010) recommended a nonlinear model for onion mass determination based on length as *M* = 0.035*a*^2^ − 1.64*a* + 36.137, *R*^2^ = 0.96.

#### Modeling based on areas

Among the investigated models based on projected areas (*PA*_1_, *PA*_2_, *PA*_3_, and *CPA*), Quadratic model of the *CPA* (Table 2) had the highest value of *R*^2^:


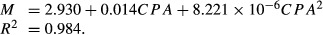
(11)

However, if this model is used for grading the fig fruits, all the three projected areas of fruit will be required. Therefore, the speed of the processing will be decreased and the costs of sorting and grading will be increased. It is evident that one of the projected areas must be selected. Among the *PA*_1_, *PA*_2_, and *PA*_3_ projected areas, Quadratic model of *PA*_1_ was preferred because of the highest value of *R*^2^:


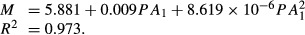
(12)

For mass prediction of the fig fruit based on surface area, the best models were Linear and Quadratic with *R*^2^ = 0.908:


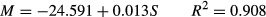
(13)


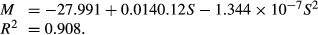
(14)

However, this model requires the measurement of three dimensions of fig fruit for geometric mean diameter (*D*_g_) and surface area (*S*), which makes the grading mechanisms more tedious and expensive.

#### Modeling based on volumes

According to the results, for mass prediction of the fig fruit based on volumes (*V*_m_, *V*_osp_, and *V*_ellip_; Table 2), the Linear model based on volume of the fruit assumed as oblate spheroid shape (*V*_osp_) with *R*^2^ = 0.965 was the best model:


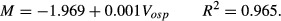
(15)

According to the results obtained in this study, the Quadratic model could predict the relationships between the mass and some physical properties of fig fruits with proper values of coefficient of determination. Finally, the Quadratic model based on the first projected area (*AP*_1_) for mass predication of fig fruits is suggested because it needs one camera, as the main part of the grading systems and it is applicable and an economic method.

## Conclusions

The results of this study can be concluded as follows:

- In this study, some physical properties of fig fruits and their relationships with fruit mass were presented. All considered properties were statistically significant at 1% probability level.
- The best model for fig fruit mass predication among the dimensional properties was Quadratic form based on width (*W*) of fruit: *M* = 58.443 − 3.318*W* + 0.064*W*^2^, *R*^2^ = 0.969.
- The best model for mass prediction of fig fruit based on three projected areas was Quadratic form based on first projected area (perpendicular to *L* direction of fig): 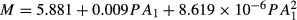, *R*^2^ = 0.973.
- Linear model based on the volume of the fruit assumed as oblate spheroid shape (*V*_osp_) with *R*^2^ = 0.965 was the best model for mass prediction of the fig fruit based on volumes: *M* = −1.969 + 0.001*V*_osp_, *R*^2^ = 0.965.
- At last, from economical standpoint of view, mass model of fig fruit based on the first projected area is recommended for designing and development of grading systems.
